# Achieving micron-scale plasticity and theoretical strength in Silicon

**DOI:** 10.1038/s41467-020-16384-5

**Published:** 2020-05-29

**Authors:** Ming Chen, Laszlo Pethö, Alla S. Sologubenko, Huan Ma, Johann Michler, Ralph Spolenak, Jeffrey M. Wheeler

**Affiliations:** 1 Laboratory for Nanometallurgy, Department of Materials Science, ETH Zürich, Vladimir-Prelog-Weg 5, 8093 Zürich, Switzerland; 2 Laboratory for Mechanics of Materials and Nanostructures, Empa, Swiss Federal Laboratories for Materials Science and Technology, Feuerwerkerstrasse 39, 3602 Thun, Switzerland; 3 Laboratory for Transport at Nanoscale Interfaces, Empa, Swiss Federal Laboratories for Materials Science and Technology, Ueberlandstrasse 129, 8600 Dübendorf, Switzerland

**Keywords:** Materials for devices, Structural properties, Synthesis and processing

## Abstract

As the backbone material of the information age, silicon is extensively used as a functional semiconductor and structural material in microelectronics and microsystems. At ambient temperature, the brittleness of Si limits its mechanical application in devices. Here, we demonstrate that Si processed by modern lithography procedures exhibits an ultrahigh elastic strain limit, near ideal strength (shear strength ~4 GPa) and plastic deformation at the micron-scale, one order of magnitude larger than samples made using focused ion beams, due to superior surface quality. This extended elastic regime enables enhanced functional properties by allowing higher elastic strains to modify the band structure. Further, the micron-scale plasticity of Si allows the investigation of the intrinsic size effects and dislocation behavior in diamond-structured materials. This reveals a transition in deformation mechanisms from full to partial dislocations upon increasing specimen size at ambient temperature. This study demonstrates a surface engineering pathway for fabrication of more robust Si-based structures.

## Abstract (web summary)

Silicon is thought to be brittle, which limits its mechanical application in devices. Here, the authors lithographically fabricate silicon and show its superior surface quality leads to near ideal strength and micron-scale plasticity.

## Introduction

The natural abundance and electronic properties of silicon make it the preeminent material in microelectronics^1^. Its high strength also makes it suitable as a structural material for microelectromechanical systems (MEMS), and its electronic characteristics make it the primary functional component for electronic devices. However, the intrinsic brittleness of Si often leads to catastrophic failures in these structural components^2^. The reliability of MEMS gyroscopes could be notably improved by increasing the failure strength of the structural Si to resist shock loading in mobile phone applications^3^. The fracture of Si at low strains also restricts the potential for strain engineering^4,5^ of its electronic properties in the elastic regime. In metal-oxide-semiconductor field-effect transistors, the carrier mobility is significantly enhanced in the Si channel by applying a uniaxial compressive strain^4,6^. Although extreme elastic strains can be achieved in grown Si nanowires (NWs) below 100 nm^7^, Si channels used in microdevices are commonly much larger (hundreds of nanometers) and fabricated by top-down etching^8^.

Notable plasticity in bulk Si has only been observed either at high temperatures (≥0.6 *T*_melt_)^2^ or under a confining pressure to suppress fracture^9^. Micromechanical tests, e.g. nanoindentation and microcompression, have also been performed to study the plastic deformation and corresponding defects at the nano-scale at low temperatures^10^. These results revealed a strongly size-dependent strength and also a brittle-to-ductile transition (BDT) at ambient temperature by reducing the specimen size down to the nanoscale^11,12^. The enhanced strength and improved plasticity were attributed to the depletion of dislocation sources in the reduced volume, which required additional stress for internal dislocation nucleation^13^ and/or free surface-facilitated dislocation nucleation^14,15^. Therefore, the effect of the surface on defect activities and corresponding deformation behaviors is significant at small volumes^16–18^.

One typical issue with microcompression testing is the effect of surface damage from focused ion beam (FIB) milling on the mechanical properties. Previous investigations have shown that subsequent thermal annealing after FIB milling effectively increased the strength of Si by alleviating FIB damage on the surface^19^. Studies employing conventional lithography techniques in semiconductors^20^ also indicated an improvement in strength over FIB-milled specimens but induced a fluorosilicate layer and/or oxide shell^21^. These additional layers significantly affect the mechanical properties and dislocation nucleation and dissipation at the surface^17^. Both FIB machining and lithography etching are routinely used in semiconductor industry and nanofabrication^22^. Nevertheless, the effect of these fabrication techniques on the surface state and resulting mechanical properties is not fully understood.

In this study, high-resolution lithographic etching is employed to fabricate Si pillars with various diameters from nano- to micron-scales. After plasma etching, a wet cleaning step is performed to remove deposited fluorocarbon residues from the surface of the pillars. Then, a thermal oxide layer is grown into the substrates and then removed by immersion into HF. This achieves a near-pristine surface state without damage or additional layers (full procedural details are given in the “Methods” section). We show lithographically-etched Si exhibits enhanced elasticity and plasticity extending into the micron-scales at ambient temperature, i.e., one order of magnitude larger than previously observed in FIB-machined samples. Moreover, Si fabricated by this lithography process also acquires strengths on the order of the theoretical strength. This suggests that significant performance enhancements might be achieved for Si-based micro-scale structures and functional devices by this surface engineering. Furthermore, the enhanced plasticity at larger scale reveals a transition from full to partial dislocations with increasing size. Our study indicates that the deformation mechanisms in Si are highly dependent on the size-dependent stresses, in addition to the normal temperature-dependent BDT. Similar behavior is expected in other semiconductor materials with diamond-cubic and zincblende structures.

## Results

### Microcompression of lithographic and FIB-machined Si pillars

**Fig. 1:**
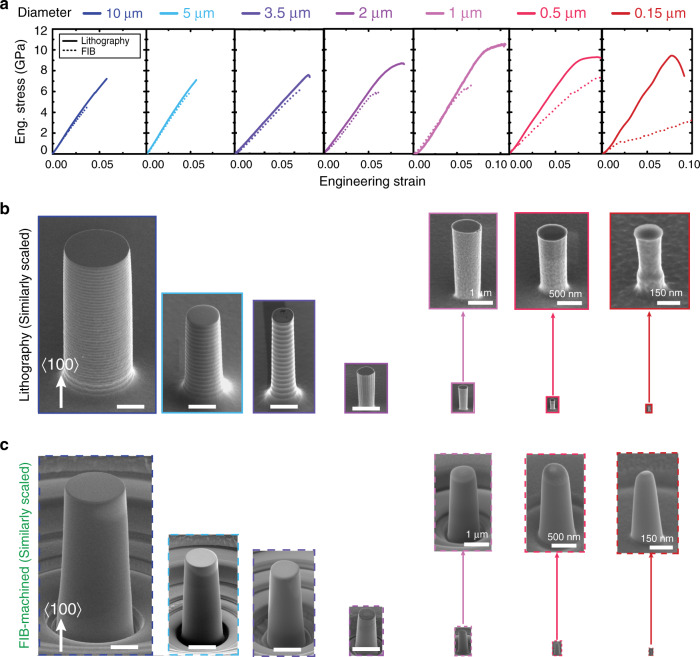
Microcompression of Si pillars fabricated by lithography processing and focused ion beam (FIB) machining at ambient temperature. **a** Representative flow curves of lithographic (solid lines) and FIB-machined (dashed lines) pillars under a constant strain rate of 5 × 10^−4^ s^−1^. **b** Similarly scaled secondary electron (SE) images of pillars with various diameters fabricated by lithography technique prior to microcompression. **c** Similarly scaled SE images of FIB-machined pillars with various diameters. Scale bar, 3 μm.

To investigate the effect of sample size and surface state on deformation behavior, Si pillars with various diameters (*D*), in a wide range of 0.15−10 μm, were fabricated by two methods: lithography etching and FIB milling. Pillars produced by lithography etching with surface cleaning are shown to display a smooth surface and a straight sidewall with minimal taper. The morphologies of pristine pillars prior to deformation are shown on a uniform scale to illustrate the wide range of sizes investigated (Fig. 1b, c). The geometries and corresponding patterning methods of each diameter are listed in Supplementary Table 2. The variation in engineering stress−strain curves between pillars fabricated by both methods over nearly two orders of magnitude in length scale is shown in Fig. 1a. As expected, increasing amounts of plasticity are observed in both types of pillars with decreasing pillar diameter. Virtually no plasticity is seen in Si pillars with diameters of 10 and 5 μm, but lithographic pillars display higher strengths compared to their FIB-milled counterparts. Surprisingly, at sizes as large as 3.5 μm diameter, lithographically processed Si exhibited plasticity, in which 0.3% engineering plastic strain was achieved at micro-scale.

In contrast, all FIB-milled specimens with diameters greater than 0.5 μm failed by catastrophic fracture without measurable plasticity (Fig. 1a). When pillar diameters were decreased to the nanoscale, i.e., with diameters of 150 and 500 nm, plasticity was observed in both types of pillars. Nevertheless, pillars from FIB milling displayed a notable softening behavior as a deviation from linear-elastic region of 〈100〉 crystalline orientation, as compared to the stress−strain behavior of the lithographic counterpart. This phenomenon is consistent with a composite behavior of the inner crystalline silicon and the amorphized FIB-damage layer, as observed previously^19,23^. Furthermore, the strength of lithographic pillars progressively increases with reducing diameter, whereas this tendency is less pronounced in FIB-machined pillars. The strength of FIB-machined pillars only effectively increases until the onset of composite behavior at the submicron scale. Lithographic pillars display superior strength, and the difference between lithographic and FIB-milled pillars becomes more pronounced at smaller sizes. This increase in strength with decreasing size is commonly referred to as the size effect. However, in this case, examination of Fig. 1a shows that this size effect does not perfectly follow a power-law trend with size as commonly described. A quantitative analysis of size-dependence of strength in this material is presented in a later section.

### Size-dependent transition of dislocation mechanisms in Si

**Fig. 2:**
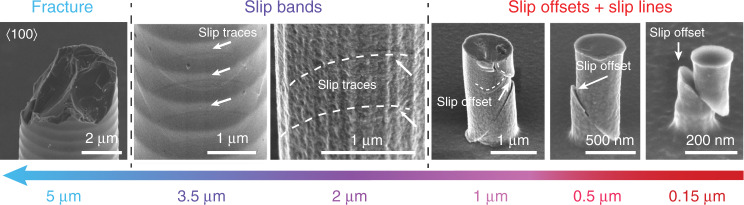
Size-dependent transition in Si deformation. Scanning electron micrographs of surface morphologies of lithographic Si pillars compressed at ambient temperature with a diameter from 5 to 0.15 μm (tilt view angle 54°).

As adumbrated in the previous section, the deformation behavior of the Si pillars varies with size. Figure 2 illustrates the size-dependent deformation morphologies of lithographic pillars after compression at ambient temperature. The fractured surface of 5 and 10 μm diameter pillars demonstrates purely brittle failure: pillars deformed elastically and then catastrophically fractured. Upon decreasing the pillar diameter, 2 and 3.5 μm diameter pillars were plastically deformed with a stack-of-cards morphology (parallel arrows) at the side surfaces. This non-persistent glide band was also observed at low temperatures in GaAs and InSb with the zincblende structure^24,25^. These bands contain extended multilayers of intrinsic stacking faults and/or twins, which results from the glide of Shockley partial dislocations with Burgers vector *a*/6〈112〉 on the {111} close-packed planes^26^. This similarity in deformation morphology suggests the operation of partial dislocations in micro-scale Si at room temperature.

Upon further decreasing the pillar diameter to the nanoscale, i.e., *D* ≤ 1 μm, the lithographic pillars displayed entirely different deformation morphologies: sharp slip offsets and slip lines at the side surfaces, similar to the deformation behavior observed in GaAs and InSb at high temperatures. The magnitude of these slip offsets (~200 nm) necessitates that hundreds of full dislocations (Burgers vector ~0.384 nm) must have been nucleated and emitted from a single source on the same {111} plane. In the smallest, 0.15 μm diameter pillars, only a single 1/2$\left\langle {1\bar 10} \right\rangle${111} slip system was activated, whereas multiple slip systems were triggered in the larger 0.5 and 1 μm diameter pillars, resulting in a few interactions. The interaction between dislocations from multiple slip systems induced notable strain hardening after yielding in the flow curve of 1 μm pillar (pink solid curve) shown in Fig. 1a. In addition, the upper portion of the 1 μm pillar shown sheared down to slightly touch the substrate during compression. This might also have partially contributed to the observed strain hardening.

**Fig. 3:**
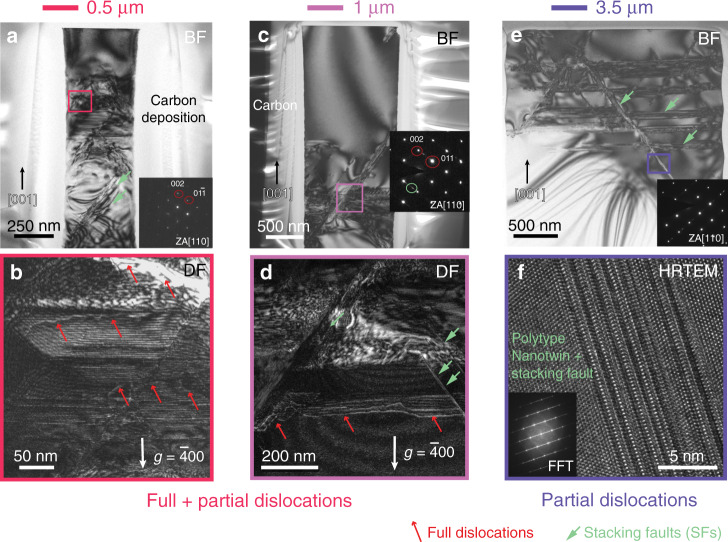
TEM analyses of FIB lamellae prepared from lithographic pillars of different diameters deformed at ambient temperature. The present micrographs of all pillars were acquired in the orientations close to [110] zone axis of an Si pillar area. Images (**a**, **c**, **e**) show regions acquired in the exact [110] zone axis orientation of different size pillars embedded into an FIB deposited protective amorphous carbon cover shell. **a** Bright-field (BF) image of 0.5 μm diameter pillar compressed to 7% engineering strain. Stacking faults (green arrows) are observed at local area. **b** Weak beam dark-field (WBDF) image acquired with *g* = $\bar 400$ shows full dislocations (red arrows). **c** BF image of a lamella cut from 1 μm diameter pillar compressed to 8% engineering strain. Twin reflections and streaks in the corresponding diffraction pattern (inset) confirms the occurrence of stacking faults (SFs) and twins. **d** WBDF image taken with *g* = $\bar 400$ shows full dislocation traces. **e** BF image of 3.5 μm pillar compressed to 6% engineering strain. **f** High-resolution transmission electron microscopy (HRTEM) micrograph of nanoscale twins and stacking faults inside the glide bands. This reveals structural ordering of SFs resulting in the formation of polytypes and nanotwins (see Fourier-transformation pattern (insert)).

In the larger pillars (*D* ≥ 3.5 μm), a scalloped sidewall is observed on the side surface. These scalloped profiles were induced by the Bosch process, during which the etching process was switched back and forth between etching using SF_6_ for a few seconds and then passivating the sidewall using C_4_F_8_ for a few seconds. After this dry etching, an oxide layer with a 2 µm thickness was thermally grown into the larger pillars and then wet etched away to smooth the sidewall scallops and consume any process-related damage and residual contamination on the pillar surface. In smaller pillars (*D* ≤ 2 μm), top-down etching was conducted using a combination of SF_6_ and C_4_F_8_ gases without alternating between them. Therefore, there is no scalloped profile on the sidewalls of the smaller pillars. After that, an oxide layer was also grown into these pillars, but with a significantly lower thickness of 10 nm, which was similarly removed afterwards. The side surfaces of all lithographic pillars are damage-free, smooth and clean, without any sharp steps or flaws, especially in smaller pillars. The observation of the deformed microstructures (Fig. 2) show that slip traces are randomly distributed in both the concave and convex parts of the scalloped surface of the 3.5 μm pillar. In the following TEM studies (Fig. 3e), horizontal glide bands were also observed to be arbitrarily distributed throughout the cross section of this pillar. It is believed that the sidewall scalloping and surface roughness have a minor effect on dislocation nucleation during plastic deformation.

High-resolution transmission electron microscopy, using both TEM and scanning TEM (STEM) modes, further revealed the nature of these size-dependent deformation mechanisms. The microstructures of the deformed 0.5, 1, 2 and 3.5 μm diameter lithographic pillars are presented in Fig. 3 and Supplementary Figs. 1‒3. As shown in Fig. 3a, numerous slip traces are more homogeneously distributed in the 0.5 μm pillar, which is surrounded by protective carbon deposited during TEM lamella preparation. These slip traces resulted from the motion of dislocations on the {111} close-packed planes in diamond-structured Si^11,27^. According to the weak beam dark-field (WBDF) image (Fig. 3b) acquired with *g* = $\bar 400$ into the [110] zone axis, full dislocations with Burgers vectors of *a*/2〈110〉, present as bright lines (red arrows), are observed extensively in these slip traces. However, another type of defect with entirely different features was also observed: a band (green arrows) with a straight boundary to the matrix in the BF image (Fig. 3a and Supplementary Fig. 2a) with the same tilt angle. These glide bands are planar defects, such as stacking faults induced by Shockley partial dislocations with Burgers vectors of *a*/6〈112〉, with a width of tens nanometers. Actually, it was rather challenging to set up weak-beam diffraction conditions in the small volumes of the 0.5 μm pillars, because the region of interest in the micropillar was constricted to small local areas.

The full dislocation mechanism was also dominant in the plastic deformation of the larger, 1 μm diameter pillar (Fig. 3c), which manifested as bright contrast from full dislocation lines in the WBDF micrograph (Fig. 3d), acquired close to [110] zone axis with *g* = $\bar 400$. In addition, more glide bands were also observed after deformation. These glide bands were nanoscale twins and/or stacking faults, which were confirmed by the paired spots (in green circle) and streaks in the corresponding diffraction pattern (Fig. 3c inset). The observation of nanotwins and stacking faults further confirmed the operation of Shockley partial dislocations on the {111} glide-set. This dissociation of full dislocations into partials was previously only considered to occur at elevated temperatures (~500−700 K) in Si^28^, because the motion of partials between narrow {111} glide-set requires high stresses at low temperature^23^. However, the present study reveals that the activation of partials is also possible at room temperature in micro-scale specimens with sufficient strength (Fig. 1a). Hence, the defect transition in the diamond structure is also stress-dependent in addition to or rather-than temperature-dependent.

Partial dislocation activity was more pronounced during plastic deformation at larger pillar sizes. The deformed microstructure of a 2 μm diameter, lithographically-processed pillar revealed both straight glide bands and curved full dislocations indicating a mixed mode of full and partial dislocations, shown in Supplementary Fig. 3. A high density of glide bands suggests that the partial dislocation activity was promoted at larger sizes. As pillar diameter increased to 3.5 μm, the contrast from full dislocation lines was no longer observed, whereas glide bands became more noticeable with greater thickness and uniform contrast across the whole pillar as shown in Fig. 3e. The high-resolution lattice image (Fig. 3f) of the glide band area shows that nanoscale twins have a thickness of several atomic layers of atoms embedded into the stacking faults. The corresponding fast Fourier-transformed (FFT) pattern (Fig. 3f inset) exhibits streaks decorated by twin reflections, confirming the coexistence of both twins and stacking faults within glide bands. The combination of twins and stacking faults indicates a polymorphism of planar defects induced by partial dislocations during plastic deformation of Si at the micro-scale. In general, the deformation mechanism in Si gradually transitioned from full dislocations being preferred at the nanoscale to a mixture of full and partial dislocations at intermediate sizes and finally to partial dislocations dominating at pillar diameters ≥3.5 μm.

### Superior mechanical properties and minor size effect in lithographic Si

**Fig. 4:**
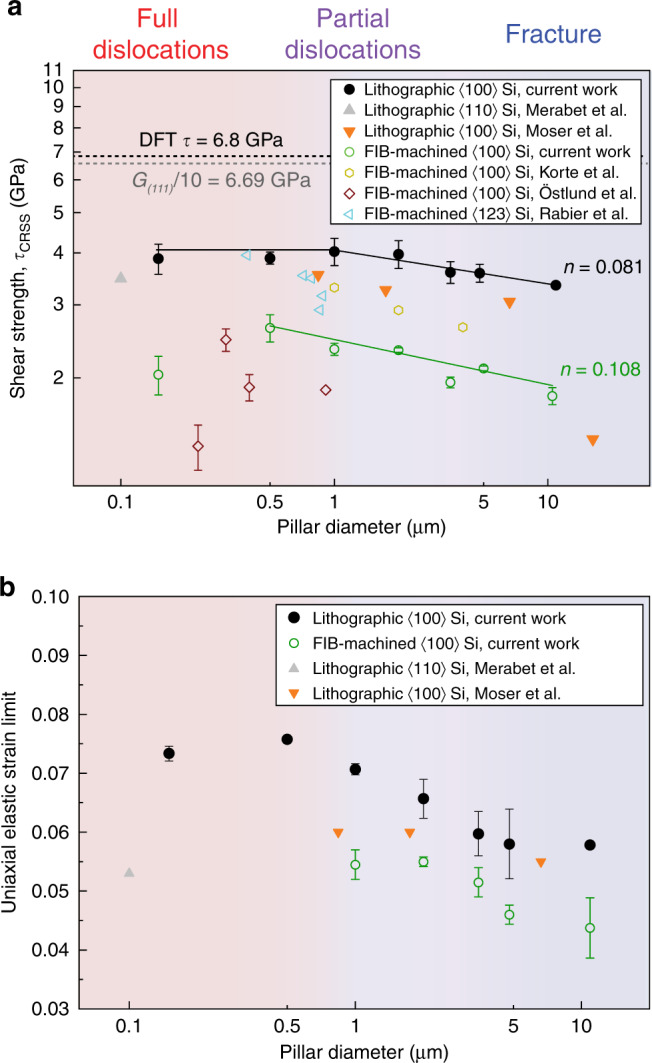
Mechanical properties of lithographic and FIB-machined Si micropillars from microcompression at room temperature. **a** Critical resolved shear stress (CRSS) as a function of pillar diameter with comparison to literature values^11,20,23,28,31,32^. The upper limit of ideal strength is calculated from density function theory (DFT). **b** Elastic strain limit as a function of pillar diameter under microcompression^11,20^. The error bars are two standard deviations.

In addition to the size-dependent dislocation mechanisms, the strength of Si was also found to vary with length scale at ambient temperature. The difference in strength between Si pillars fabricated by lithography and FIB milling processes increased with decreasing diameter. Moreover, lithographic pillars displayed 50−85% higher strength than the FIB-machined pillars with a comparable diameter over the whole range of diameters, as shown in Fig. 4a. The shear strength (*τ*_CRSS_ = ~4 GPa) of lithographic Si pillars at all sizes exceeded a large fraction of shear modulus of Si to approach the ideal strength of Si at ambient temperature (*G*_*(111)*_/10 = 6.69 GPa, where *G*_*(111)*_ is the shear modulus of (111) crystalline plane)^29^, labeled in Fig. 4a. Density functional theory predictions suggest a theoretical maximum shear strength for Si of 6.8 GPa or ~*G*/9, though deviation from linearity, or yielding, is observed in their calculated stress−strain curves at shear stresses >4.5 GPa^30^. The strength of lithographic Si pillars in the present study is the highest ever measured in nanomechanical tests with comparable sample sizes^11,20,28,31,32^.

Considering the relationship of size and strength, the effect of specimen size on the *τ*_CRSS_ of materials is usually quantified in terms of a power law^33^:

1 $${\uptau}_{CRSS} = C \cdot D^{ - {{n}}},$$

where *C* is a fitting parameter, *D* is the size of sample, and *n* is termed the size effect exponent or strengthening exponent. By taking the natural logarithm on both sides of Eq. (1), the correlation between *τ*_CRSS_ and *D* is expressed as follows:

2 $${\mathrm{ln}}({\uptau}_{{\mathrm{{CRSS}}}}) = {\mathrm{ln}}({\it{C}}) - n \cdot {\mathrm{ln}}\left( {\it{D}} \right).$$

Equation (2) expresses a linear relationship between ln(*τ*_CRSS_) and ln(*D*), from which the power law exponent *n* of Si is observed as the slope—labeled in Fig. 4a. The critical resolved shear stress (CRSS) of lithographic pillars was only fitted until 1 μm diameter, below which the shear strength displayed a plateau (~4 GPa) as a function of the diameter. The calculated slopes indicate a relatively weak size effect in Si at room temperature with values of *n* in the range of 0.08−0.11, with lithographic Si pillars displaying a smaller value (*n* = 0.08) than FIB-milled pillars (*n* = 0.11). These values are much lower than single crystalline *bcc* metals, such as W and Ta (*n* = 0.3−0.4)^34^, and *fcc* metals like Cu and Ni (*n* = 0.61−0.97) in similar homologous temperature (*T/T*_melt_) ranges^35^. However, this small size effect is in good agreement with values from covalent crystal of GaAs (*n* = 0.08−0.12)^36^ and ionic crystals of LiF (*n* = 0−0.1)^37^ and MgO (*n* = 0.1−0.3)^38^ with sizes at micro-scales, as well as metallic NWs with diameters below 100 nm at nano-scales^39^.

The weak size effect in Si can be explained if the surface nucleation of defects remains the dominant deformation mechanism even at larger length scales, i.e., the micron-scale. In *fcc* and *bcc* metals at the micron-scale, the size effect is commonly attributed to two mechanisms: the single-arm source model by Parthasarathy et al.^40^ and the dislocation exhaustion/surface nucleation model by Greer et al.^33^ However, surface nucleation-mediated plasticity is only considered in the case of dislocation-free whiskers or etched pillars in metals at nanoscale^17,41^. In metals, dislocation densities are normally^42^ of the magnitude of 10^12^−10^14^ m^−2^, which makes the number of single-arm dislocation sources inside metallic micropillars in the range of 10^2^−10^4^. Thus, the dislocations can be alternatively propagated either from preexisting dislocation sources in the volume or by free surface nucleation. In contrast, in Czochralski-grown semiconductor crystals^43^, the dislocation density is reduced to below 10^8^ m^−2^. This reduces the probability of a preexisting dislocation source inside an Si micropillar to between 0.01 and 1%. This requires dislocations to be nucleated to enable deformation. However, dislocation nucleation from the free surface is more energetically favorable than homogeneous nucleation in volume^44^, because surface steps and drawbacks on the surface effectively facilitate the nucleation process by inducing stress gradients^14,45^. It is supported by molecular dynamics (MD) simulations of atomic-scale deformation mechanisms in Si pillars and NWs by Brochard et al.^45^. The nucleation-mediated deformation behavior in NWs has been shown to be significantly affected by the surface state from fabrication process but only weakly affected by size.^17^ Thus, the size effect is smaller in semiconductors with a purely surface nucleation mode compared to the volume-dependent theory of single-arm sources. Small size effects in Si are also attributed to its high Peierls’ stress at ambient temperature in contrast to most metals. The lattice resistance of covalently-bonded Si is in the range of 0.1−0.5*G* (*G* being the isotropic shear modulus), which is three orders of magnitude higher than metals (~10^−4^*G*)^34,46^. The movement of dislocations is considerably hindered by such high lattice friction, even though the nucleation of dislocations is promoted in pillars at micron-scales. This dependence of size effect on lattice resistance has also been observed in ionic crystals, i.e. MgO and LiF, as well as *bcc* refractory metals and high entropy alloys^47^ (with high Peierls’ stress for metals) below their critical temperatures.

In addition to high strength, our micron-scale lithography Si pillars also achieved ultrahigh elastic strain limits in the range of 6−7.5% (Fig. 4b). The uniaxial elastic strain of our Si specimens is ~20−40% higher than pillars made by either conventional lithography etching or FIB machining. This elastic strain limit is much higher than previous top-down etched Si NWs (~4%)^48^ and comparable to most grown Si NWs with a near-pristine crystalline structure^7^. Ultrahigh elastic limit (~16%) and high strength (20 GPa) were achieved in Si NWs grown by the vapor−liquid−solid (VLS) process^48^. However, the present lithographic Si pillars exhibited superior compressive ductility, i.e. 3% plastic strain in 0.5 μm diameter pillars, in contrast to the catastrophic brittle failure observed during tensile loading of VLS-grown NWs. This suggests Si may have significant tensile/compressive plastic asymmetry. In microdevice manufacturing, top-down etching is much more extensively used than growth processing for the fabrication of Si-based building blocks. Our results indicate that Si processed by lithographic etching with an appropriate surface cleaning procedure demonstrates far superior elasticity. This allows functional performance to be further enhanced by imposing larger elastic strains.

## Discussion

**Fig. 5:**
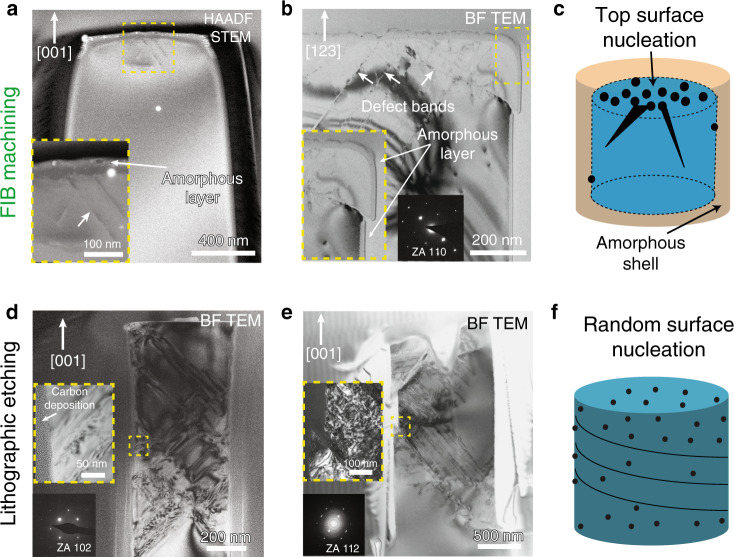
Different deformation microstructures of lithographic and FIB-machined Si pillars. **a** High-angle annular dark-field (HAADF) STEM micrograph of lamella cut from 〈100〉-oriented pillar with 1 μm diameter from FIB milling after deformation (*ε* ~ 4% engineering strain) with defect bands initiating from the pillar apex covered by an amorphous layer. **b** BF TEM image of lamella cut from 〈123〉-oriented pillar with 1.5 μm diameter from FIB milling (*ε* ~ 5% engineering strain) with more defect bands and cracks initiating from the top of the pillar. **c** Schematic illustrations of dislocation nucleation sites and crack initiation at the top interface of FIB-milled pillar with amorphous shell. **d** BF TEM micrograph of lamella cut from lithographic 0.5 μm diameter pillar after deformation (*ε* ~ 10% engineering strain) with homogeneous distribution of slip traces. The outside carbon was deposited during TEM lamella preparation (inset). **e** BF STEM image of a lamella cut from 1 μm diameter pillar compressed to 10% engineering strain. **f** Schematic of random nucleation sites of dislocations at free surface in lithographic etched pillar.

In the present study, lithographic Si pillars exhibited superior mechanical properties, enhanced elasticity and plasticity coupled with higher strength, compared to FIB-machined pillars. FIB-milled pillars acquired an amorphized layer at the surface (Fig. 5a, b) with a thickness of ~40 nm (Supplementary Fig. 4c). The influence and relative volume fraction of this amorphous layer became more significant with decreasing size to nanoscales: the volume fraction of the amorphous layer increased 75% in the 0.15 μm diameter pillar. Since the modulus of amorphous Si (*E* = 33.3 GPa)^19^ is much lower than 〈100〉-oriented crystalline Si (*E* = 127 GPa)^49^, a decrease in modulus was observed in FIB pillars with a large amorphous volume fraction due to the composite behavior of the amorphous shell and crystalline core of the pillar (Fig. 1a, red dotted curves). This amorphous fraction also caused the strength of the FIB-milled nanopillars to deviate from the power law trend (Fig. 4a), so these sizes were ignored in the subsequent determination of the size effect exponent, *n*.

Furthermore, the deformed microstructures in pillars from FIB milling and lithographic processing show striking differences. FIB-milled pillars were only compressed to 4‒5% engineering strain to preserve the deformed microstructure and avoid catastrophic failure. In FIB-milled pillars with a 〈100〉 orientation, defects were observed to exclusively initiate from the top of the pillar, shown in Fig. 5a. In 〈123〉-oriented pillars from similar FIB milling, these glide bands and even cracks propagated further downward into the pillar at the slightly higher engineering strain of 5% (Fig. 5b). We attribute this localization to the amorphous layer and sub-surface defects introduced by the FIB. The top surface of FIB-machined pillars was exposed to direct, 90° incidence Ga^+^ irradiation due to beam tails. Although this dose of Ga^+^ is minor, ions impinging at 90° incidence have high momentum^50^, and these implanted defects can act as easy nucleation sites for dislocations and lower the stress required for deformation^50,51^ (Fig. 5c).

However, lithographic pillars display homogeneously-distributed dislocations (Fig. 5d, e), indicating random nucleation of dislocations from the free surface of the pillars. This uniform deformation sharply contrasts with the localized deformation seen in the FIB-machined pillars, where cracks and defects are concentrated at the top part of pillar. The high magnification image at the sidewall of a lithographic pillar (Fig. 5d inset and Supplementary Fig. 1a) shows a straight interface between the crystalline Si pillar and the amorphous carbon deposited during lamella preparation, which is further confirmed by EDX elemental mapping (Supplementary Fig. 1b). This indicates that the surface of Si after the lithography and cleaning cycle is clear and regular without the amorphous surface layer observed in FIB-machined pillars. In the lithographic 1 μm pillar, the high magnification DF image (Fig. 5e inset) showed a large number of full dislocations forming a network adjacent to the sharp slip offsets observed in SE observation of the 1 μm diameter pillars (Fig. 2). Pillars made by the present lithographic process preserved their low defect density and display a pristine surface with only a ~1 nm native oxide layer^52^. The improved surface quality overall prevents early dislocation nucleation and multiplication from defects and effectively postpones the initiation of dislocation events, thereby extending the elastic regime^48^. Without the easy nucleation sites seeded in by FIB-machining, additional stress was needed to randomly nucleate dislocations at the free surface of lithographic pillars to enable plastic deformation (Fig. 5f). Consequently, the lithographic pillars display superior strength, approaching the theoretical ideal stress of Si (Fig. 4a).

In addition to greater strength, lithographic Si pillars also attained enhanced plasticity, extending the nominal brittle-to-ductile transition (BDT) size from the nano- to the micron-scale. We attribute this increased ductility to the delocalization of deformation away from easy FIB-implanted defects and towards the larger number of surface sites for dislocation nucleation. In comparison, plastic deformation was localized at the damaged pillar top (with a taper observed) as a concentration and interaction of glide bands and cracks (Fig. 5a, b). The greater number of nucleation sites in lithographic pillars allows for higher nucleation rates, sustaining plastic deformation at higher strain rates and/or larger sizes. This allows the length scale for such surface nucleation-dominated behavior to be extended into the micron-scale. The nucleation rate $(\dot n)$ of dislocations per unit volume at given temperature (*T*) is written as: $(\dot n) = \dot n_0\exp ( - (U - \tau V)/kT)$^44^, where $\dot n_0$ is the attempt frequency of nucleation event, *U* is the activation energy of dislocation nucleation in Si, which is about of 1−5 eV^53^ depending on the exposed strain (in the range of 0.6−0.7, Fig. 4b), *τ* is the applied stress on the activation volume of dislocation motion, *V*, in the range of 1−10 *b*^3^ for the surface nucleation^54^, and *k* is the Boltzmann constant. This shows that increases in stress, *τ*, result in an exponential increase in nucleation rates, $\dot n$, so the higher stresses required to nucleate from the surface sites also significantly promote greater nucleation rates and thus induce enhanced plasticity. This high stress coupled with promoted plasticity was also recently observed in microcompression of Mg with limited ductility at large scales.^55^ Furthermore, the amorphous surface layer in FIB-machined pillars hinders surface self-diffusion for dislocation nucleation and increases the required activation energy and activation volume^56^. The incoherent interface between crystalline core and amorphous shell also constricts the escape of dislocations at the surface, which then generates a back-stress which induces dislocation pile-ups and crack formation^57^.

The wider range of sizes where plasticity is observed in lithographic Si pillars reveals a stress- and/or size-dependent transition from full to partial dislocation-mediated plasticity at ambient temperature. Traditionally, this transition was only observed at elevated temperatures in diamond-structured materials^2,24^. In this work, we observe this transition at ambient temperature by using only the size effect to vary the stress. This observation might be explained by considering two stages of plastic deformation: dislocation nucleation and subsequent dislocation propagation in defect-depleted Si pillars. The defect-scarce nature and pristine surface of the pillars acts to increase the stress with decreasing size as the absolute number of possible surface sources decreases with the pillar size^16^. At small sizes, these high stresses allow the nucleation and operation of both partial and full dislocations sources (Fig. 3). According to previous experiments^27^ and MD simulations^58^, full dislocations are only seen at high stress conditions, while partial dislocations are favored at low stress, such as at high temperatures. During the dislocation nucleation process, incipient dislocation lines bulge to form kink-pairs and induce local energy perturbations. According to the line tension model of dislocation energy, the activation enthalpy of this kink-pair nucleation is equal to the dislocation line energy minus the mechanical work done by the applied stress^59^. Activation enthalpies of both full and partial dislocations decrease with increasing stress at the same temperature, but the decrement in enthalpy fraction of full dislocations with their larger Burger vector is more significant than in partials. This means the nucleation of full dislocations requires a smaller activation enthalpy than partial dislocations. Therefore, full dislocation nucleation is more energetically favored under high stress conditions due to the different configurations of full and partial dislocations in the Si lattice.

After nucleation, dislocations must overcome the Peierls’ barrier to propagate and accommodate deformation. MD simulations show that Peierls’ stress for full dislocation motion in Si, 3.8−4.5 GPa^46^, is higher than for partial dislocations, ~3.3 GPa^60^. These stress levels are in good agreement with our observed stresses in the different size regimes. Further, the dislocation-transition model in *fcc* metals^61^, which share the exact same slip system as Si, also shows that the CRSS for full dislocations is higher than for partial dislocations in Si (stacking fault energy of ~50 mJ m^−2^)^62^. However, the question arises as to why a high stress mechanism can operate when a lower stress mechanism is simultaneously available. This may be explained by the different levels of efficiency between the operations of partial and full dislocation sources. The yield stress was defined here as the stress level of the first deviation from linear-elastic behavior, which is correlated with dislocation nucleation and propagation from the surface. The stress for surface nucleation is independent of sample size^54^, so the observed increase in stress is due to the size effect on dislocation propagation: dislocation arm truncation/bowing due to dimensional constraint. This increases the stress in smaller pillars for both partial and full dislocation operation, which is seen in the increase in elastic strain limit in Fig. 4b. However, these two mechanisms compete to accommodate plastic strain. During loading, the nucleation of partial dislocations is favored at lower stress, but the stress to propagate these incipient partials is also increased by the size effect. However, partial dislocation sources typically exhaust themselves after one operation, which is insufficient to accommodate more than a few Burgers vectors of strain. In dislocation-scarce pillars, these few sources may be exhausted without accommodating obvious strain, allowing further elastic straining and stress increase until the activation of full dislocations. Full dislocation sources may operate indefinitely to emit a large amount of full dislocations from a same source and accommodate much higher levels of strain^32^, which results in a plateau of strength on their activation at small sizes (Fig. 4a). MD simulations also show that full dislocations in Si^63^ are more mobile than partials over a wide stress range. This greater efficiency and mobility is illustrated by the magnitude of the slip offsets in Fig. 2, where full dislocation slip accommodates the majority of strain in the smaller pillars, despite the concurrent deformation by partial dislocations in the same size range. Thus, the transition in partial to full dislocations results from a low efficiency of partial dislocations with an insufficient number of sources at small pillar diameters to accommodate strain, such that the stress levels increase to the point where full dislocations operate and accommodate larger levels of strain. Overall, the partials mechanism is suppressed in smaller pillars by full dislocations with higher efficiency and mobility.

To summarize, we achieved ultrahigh elastic strain limits, near ideal strength (shear strength ~4 GPa) and plastic deformation in Si at the micron-scale by using lithographic processing to obtain an undamaged, free surface state. This enhanced plasticity enabled the first observation of a size- and stress-dependent transition in dislocation behavior, where full dislocations become preferred at small sizes and high stresses. The stresses involved are consistent with previous theoretical calculations and are on the order of the ideal stress for plastic deformation. The magnitude of these stresses and the relatively minor size effect indicate that dislocation nucleation is the primary, rate-limiting deformation mechanism operating in these dislocation-scarce structures. Similar lithographic procedures for Si should enable fabrication of high strength and more robust MEMS/microdevices, as well as enhanced performance in functional components.

## Methods

### Si pillar fabrication

Si pillars were fabricated by modern lithography patterning combined with reactive ion etching (RIE)^64^ on Si single-crystal wafers (100 mm diameter, 〈100〉 orientation, P type/Boron-doped, sheet resistivity 15−25 Ohm·cm, 525 μm thickness). To study the effect of sample size on mechanical properties, Si pillars were fabricated with diameters varying from sub-micron to several microns, as listed in Supplementary Table 1. Therefore, different beam writing techniques were employed to achieve sufficient resolution of pillar patterns with various diameters during the lithographic procedure. Direct ultraviolet (UV) laser writers (VPG 200 and MLA 150, Heidelberg GmbH) were used to write the pattern of larger pillars, i.e. with diameters of 2, 3.5, 5 and 10 μm, on the photoresist. For smaller pillars, an electron-beam (e-beam) writer (Vistec EBPG5000 100 kV) with high resolution was applied to transfer the pattern of smaller pillars, i.e. 0.15, 0.5 and 1 μm, to the electron-beam-sensitive resist. After beam patterning, RIE processing was performed in a plasma etcher (Alcatel AMS 200SE) using a mixture of fluorinated gases: SF_6_ to etch silicon and C_4_F_8_ to passivate the sidewall of the etched structures. The etching method for the small pillars, i.e., *D* ≤ 2 μm, consisted of simultaneous etching and passivation^20^. For etching the large pillars, i.e., *D* > 2 μm, the gases for etching and the sidewall passivation were alternated in cycles lasting a few seconds each^64^ to achieve deep etching of Si. After etching, an additional surface cleaning was performed to remove fluorocarbon residues deposited by the plasma etch on the surface of the pillars. First, a standardized solution of ammonium hydroxide and hydrogen peroxide was used to remove the fluorocarbon residues. After that, a mixture of sulfuric acid and hydrogen peroxide was used to remove the ionic contamination introduced in the first step. Subsequently, an oxide layer was then grown onto the substrates in a wet atmospheric thermal oxidation furnace. The oxide layer growth consumes silicon from the surface, incorporating any remaining process residues or structural damage. This grown layer is finally removed by immersion of the substrates into an HF bath to obtain a smooth surface. The details of advanced lithography combined with RIE process are described in Supplementary Note 1.

To compare the different pillar fabrication techniques, another batch of pillars with nominally identical geometries to the lithographic pillars were produced on the same 〈100〉-oriented wafer as the lithography pillars and on another 〈123〉-oriented wafer using a Helios G3 Gallium (Ga^+^) FIB (Thermo Fisher Scientific, Waltham, USA) with an acceleration voltage of 30 kV. Progressively smaller milling currents were used in multiple stages to produce the pillars. A higher initial current, in the range of 2.5−9.3 nA, was used for coarse milling depending on the diameter of pillar, then a lower current of 0.79 nA was applied to dimension the pillar close to the designed geometry. A final surface polishing step was performed using a current in the range of 40−80 pA to minimize taper and achieve the final dimension. For the fabrication of the smallest, 140 nm diameter nanopillar, a current of 0.79 nA was used for coarse milling followed by a 40 pA current for final polishing.

### Micromechanical testing

Microcompression was performed on the Si pillars in situ in a Vega 3 (Tescan, Brno, Czech Republic) scanning electron microscope (SEM) operated at 5 kV, using a SEM Indenter (Alemnis AG, Thun, Switzerland) with a diamond flat punch tip (Synton MDP, Nidau, Switzerland)^65,66^. All pillars were compressed under a constant strain rate of 5 × 10^−5^ s^−1^ using intrinsic displacement control with the compression axis parallel to 〈100〉 orientation of the Si. During microcompression testing, load−displacement curves were recorded at a sampling rate of 40 Hz. These load−displacement curves were converted into engineering stress−strain curves using the diameter and height of each pillar respectively after correcting for the frame compliance and sample substrate compliance^65^. The diameter for stress calculations was taken at the middle of the pillars, as this gives the average stress and as plastic deformation was commonly observed in this region (Figs. 2 and 3). Si pillars with submicron diameters from both fabrication processes exhibited a taper angle only around 2°. This taper was slightly increased to 3‒4° in FIB-machined pillars at submicron-scales. According to previous finite element simulations, the resulting stress and strain errors from this taper should be about 1−2%^67^.

Since the larger pillars displayed linear-elastic brittle fracture, the fracture stress of brittle pillars was used to calculate the CRSS with the assumption that crack nucleation coincided with dislocation nucleation. For plastically deforming pillars, the yield stress for CRSS was defined as the stress at the first deviation from linear-elastic behavior. The uniaxial elastic strain of microcompression was determined as the strain at the fracture of brittle pillars, or the strain at the yield point of plastic pillars.

### Microstructure analysis

After microcompression, the surface morphology of the deformed pillars was imaged using a Magellan high-resolution SEM (Thermo Fisher Scientific) operated at 3 kV. For the TEM analysis of the internal microstructure in deformed pillars, FIB-lamellae were produced using a Helios G3 station (Thermo Fisher Scientific) operated at 30 kV of Ga^+^ and 5 kV of electron beam sources. During TEM lamella production, special care was taken to ensure the lamella plane contained the 〈100〉 compression axis and the 〈110〉 slip direction. In this geometry, the slip offsets and glide events were clearly observed in TEM. To minimize the FIB-induced artifacts, steadily reducing ion beam currents down to 7 pA were used for the final polishing of the lamella surface to remove damaged layers from FIB milling. The study of defect structures was performed using a Talos F200X (Thermo Fisher Scientific) operated at 200 kV in both TEM and scanning TEM (STEM) modes and instrumented with a double-tilt sample holder.

## Supplementary information

Supplementary Information

## Data Availability

The authors declare that all data supporting the findings of this study are available within the article and its supplementary information files.
