# Supplementary material for: Achieving micron-scale plasticity and theoretical strength in Silicon
Source: Nat Commun. 2020 May 29;11:2681. doi: 10.1038/s41467-020-16384-5 (PMC7260211; doi:10.1038/s41467-020-16384-5)
Supplement: Supplementary file 1 — Supplementary Information [file 41467_2020_16384_MOESM1_ESM.pdf]

Supplementary Information for

Achieving micron-scale plasticity and theoretical strength in Silicon

Chen et al.

Supplementary Note 1: Lithographic Processing.

Substrates

Czochralski-grown ⟨100⟩-oriented single crystal silicon wafers were used with a diameter of 100 mm and a thickness of 525 μm. The P-type, Boron-doped substrates were specified with a resistivity ranging from 15 to 25 Ω-cm by the supplier. Sheet resistance measurements were carried out on 49 points on each wafer. The averaged sheet resistance values per wafer ranged from 299.1 to 310.7 $\Omega\cdot sq^{-1}$, and the standard deviation for each wafer was lower than 0.7%.

0.15, 0.5 and 1.0 μm diameter pillars

The substrates were spin coated with 2 mL of hydrogen silsesquioxane (HSQ-XR1541-006, Dow Corning) e-beam sensitive resist at 3000 RPM for 45 s, resulting in a coating of 200 nm thickness. This material becomes porous silicon oxide upon drying and densifies under e-beam exposure. The dense material etches with a lower rate compared to its unexposed state during development. The pattern exposure was carried out in a Vistec EBPG5000 100kV e-beam writer with a dose of 2200 $\mu C\cdot m$^$^{-2}$. Further exposure parameters are listed in the table below (Supplementary Table 1). To enhance the sidewall contour and to compensate for the background exposure originating to a larger exposed unit area, proximity effect correction was applied during patterning of the 1 μm diameter pillars.

Table 1. Exposure parameters of e-beam writing of pillar pattern.

Pillar diameter (nm)	Grid size (nm)	Beam current (nA)	Beam diameter (nm)	Writing speed (MHz)
150	3	10	9	48
500	7	50	29	44
1000	12	150	77	44

Following exposure, the patterned material was developed in a TMAH solution (MF CD26, MicroChem Corp.) for 2 min, then rinsed in deionized water. The HSQ film was densified in an RF O_2 plasma for 2 min at 400 W to increase its resistance during the following silicon etch step. Since HSQ is negative tone, most of the substrate was unprotected during the etching. To cover this unused area of the substrate, an ultraviolet-sensitive photoresist (AZ ECI 3007, Microchemicals GmbH) was spin coated on top of the HSQ pattern with 5800 RPM speed for 45 s, resulting in 600 nm thickness. This layer was exposed with a direct laser writer (MLA 150, Heidelberg GmbH), to clear the proximity of the pillar pattern, at 405 nm and 110 $mJ\cdot cm$^$^{-2}$ dose. The ECI photoresist was manually developed in AZ400K (Microchemicals GmbH) and rinsed in deionized water.

The dry etch was performed in an Alcatel AMS 200SE plasma etcher using a combination of SF_6 and C_4F_8 gases. The 600 nm, 1.5 μm and 3 μm height pillars were etched for 27, 68 and 135 seconds, respectively. The ECI layer was then removed in an O_2 plasma. The silicon etching process left fluorocarbon deposits on the etched surface, which were removed by a surface cleaning procedure. The first bath of this surface cleaning consisted of a standardized solution of ammonium hydroxide and hydrogen peroxide; while the second step was a mixture of sulfuric acid and hydrogen peroxide. After that, an oxide layer in a wet atmospheric thermal oxidation furnace with 10 nm thickness was grown onto pillars, which smoothens the sidewalls and incorporates any process related damage and residual contamination by consuming silicon from the surface. This oxide layer and the remaining HSQ pattern were both etched away by immersion into a buffered HF bath, and then rinsing in DI water.

2 μm diameter pillars

The substrates were spin coated with the same UV-sensitive photoresist at 850 RPM for 45 s to deposit a 1.5 μm thick coating. This was exposed by direct laser writing (VPG 200, Heidelberg GmbH) at a 355 nm wavelength using optical autofocus and a 4 mm focal distance write head. This setup allows for a single exposure spot size down to 600 nm and a step size of 12.5 nm. The photoresist was developed by immersion into AZ400K and rinsed in deionized water, then it was hardened by baking at 80 °C for 4 hrs. The rest of the etching process steps were identical to the procedures described above, except for the dry etch duration being 270 s, and the grown wet oxide layer had 100 nm thickness.

3.5–10 μm diameter pillars

The substrates were spin coated with photoresist (AZ ECI 3027, Microchemicals GmbH) at 2050 RPM to obtain a coating with 1 μm thickness. The pattern was exposed by direct laser writing (MLA 150, Heidelberg GmbH) at a 405 nm wavelength, 130 mW·cm^$^{-2}$ intensity and a 1 μm spot size. The development and photoresist hardening steps were equivalent to the processes described above. The dry etch used in this case was the Bosch process in an Alcatel AMS200SE plasma etcher, which employs alternating SF_6 and C_4F_8 gases for etching and sidewall passivation. The process duration was 178 and 356 seconds for the 15 and 30 um tall pillars, respectively. The alternating steps result in a scalloped sidewall profile with a typical step width of 200 nm and a height of 600 nm. A 2 μm thick oxide layer was grown onto pillars, with the purpose of cleaning the surface and smoothening the sidewall scalloping. The rest of the etching process steps were identical to the procedures described above.

Each wafer was recoated with a 15 um thick photoresist layer (AZ9260, Microchemicals GmbH), to protect the pillars and maintain surface cleanliness during

the following wafer dicing step. A Disco DAD321 automated dicing saw was used to slice the substrates into 10x10 mm square chips. The protective coating is then dissolved by rinsing the chip in acetone and isopropanol.

Table 2. Details of Si pillars fabricated by lithography and reactive ion etching (RIE) process. Each diameter of pillar has a FIB-machined counterpart for comparison.

Diameter (μm)	Height (μm)	Aspect ratio (height/diameter)	Pattering technique
0.15	0.45	3	e-beam
0.5	1.5	3	e-beam
1	3	3	e-beam
2	6	3	UV laser
3.5	13	3.7	UV laser
5	13	2.8	UV laser
10	26	3.25	UV laser

Supplementary Note 2: Microstructural characterization of deformed pillars.

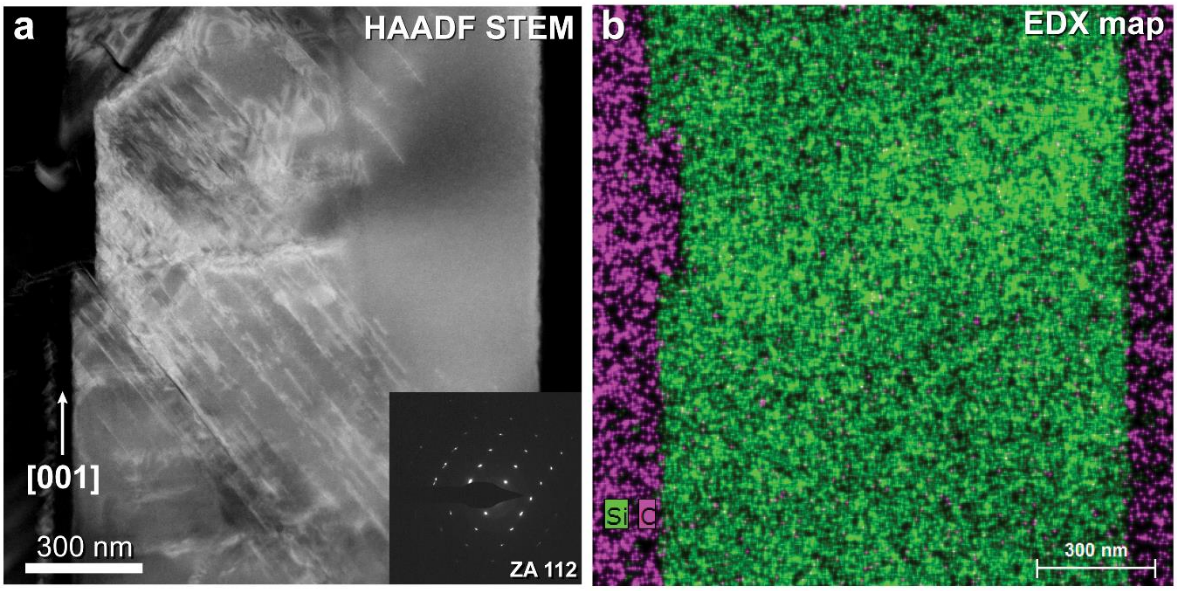

Figure 1. Microstructure of 1 μm lithographic pillar after deformation to 10% engineering strain. **a** High-angle annular dark-field (HAADF) scanning transmission electron microscopy (STEM) micrograph shows the contrast of full dislocations and stacking faults. **b** Corresponding energy dispersive X-ray (EDX) STEM elemental map indicating the clean pillar surface.

The microstructure of the lithographic pillar with 1 μm diameter after deformation is shown in high-angle annular dark-field (HAADF) STEM image (Supplementary Fig. 1a). Extensive contrast can be observed from dislocations and bands indicating substantial dislocation activity during plastic deformation, during which defects were nucleated at the surface and then propagated across the pillar. In Supplementary Fig. 1b, the EDX elemental map displays a clear and straight interface between silicon and carbon deposited during lamellae preparation. This indicates a pristine pillar surface without any additional layers after lithographic processing.

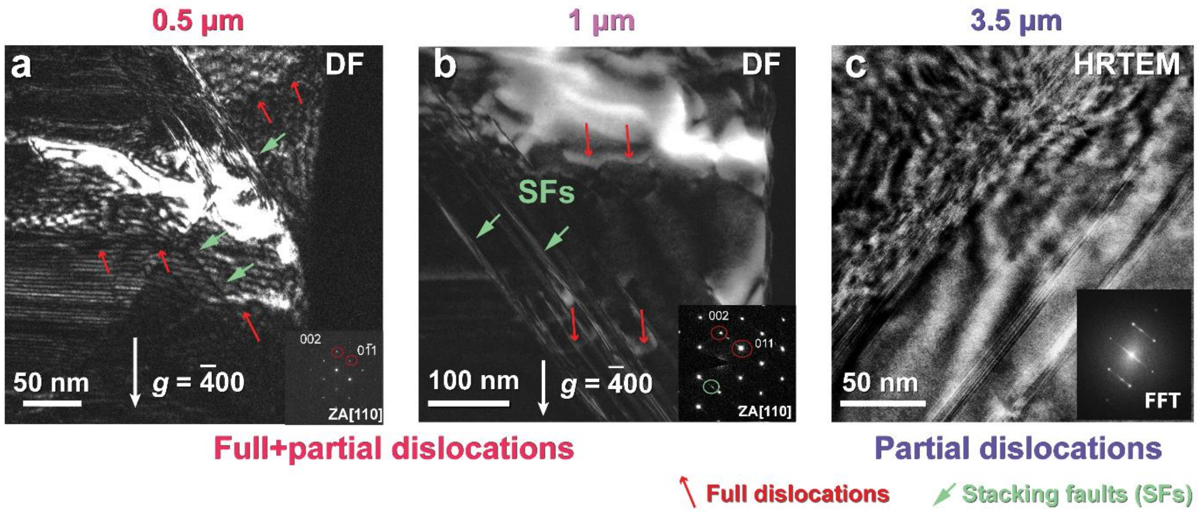

Figure 2. TEM analyses of FIB lamellae prepared from lithographic pillars of various diameters deformed at ambient temperature. All micrographs of all pillars were acquired in the orientations close to [110] zone axis of an area. **a** Weak beam dark-field (WBDF) micrograph acquired with $g = \bar{4}00$ demonstrating the interaction of full dislocations and stacking faults (green arrows) in 0.5 μm diameter pillar compressed to 7% engineering strain. **b** WBDF image acquired with $g = \bar{4}00$ shows the interaction of stacking faults with full dislocations in a lamella cut from 1 μm diameter pillar compressed to 8% engineering strain. **c** High resolution transmission electron microscopy (HRTEM) micrograph showing numerous SFs and nanotwins in 3.5 μm pillar compressed to 6% engineering strain.

In the smaller 0.5 μm pillar, full dislocations are observed in the slip traces according to the weak beam dark-field (WBDF) image (Fig. 3b) acquired with g = $\bar{4}00$ into the [110] zone axis. However, another type of defect with entirely different features was also observed: a narrow band (green arrows) with a straight boundary to the matrix in the dark-field (DF) image (Supplementary Fig. 2a) with the same tilt angle. These glide bands are planar defects, such as stacking faults induced by Shockley partial dislocations with Burgers vectors of $a/6\langle 112\rangle$, with a width of tens nanometers and interact with local full dislocations. In the 1 μm pillar, full dislocations and glide bands from partial dislocations were also concurrently observed after plastic deformation (Supplementary Fig. 2b).

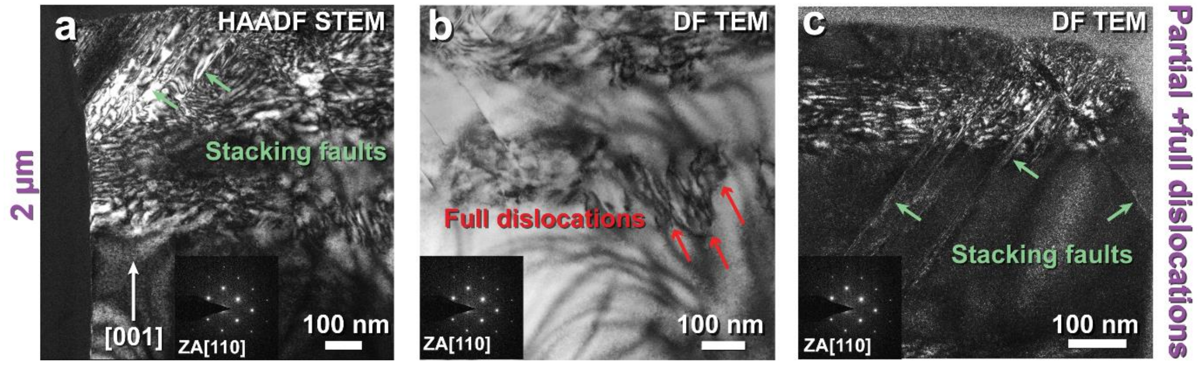

Figure 3. TEM Micrographs of lithographic pillar with a diameter of 2 μm compressed to 10% engineering strain. **a** Contrast from extensive stacking faults and curved dislocation lines randomly initiated from pillar surface. **b** Magnified dark-filed (DF) images of curved full dislocation at the local area. **c** Glide bands originating from pillar surface.

The deformation structure of 2 μm diameter lithographic pillar was also characterized in TEM, as shown in Supplementary Fig. 3. Extensive defects induced several different types of contrast in TEM micrographs. Stacking faults and curved full dislocation lines were homogeneously distributed over the whole pillar. According to surface nucleation theory, these dislocations were randomly nucleated from the surface and then propagated into pillar during plastic deformation. The curved lines of full dislocations can be observed in the magnified BF micrograph of local areas (Supplementary Fig. 3b). Moreover, glide bands with uniform contrast and a straight boundary with the undeformed matrix were also found in the DF image (Supplementary Fig. 3c). These glide bands intersected with full dislocations during propagation. Compared to the 1 μm diameter pillar, the glide bands are more extensively distributed in a large region of deformed pillar. This indicates that the partial dislocation mechanism was promoted in 2 μm diameter pillars. In the larger 3.5 μm diameter pillar, partial dislocation mechanism was further promoted as pronounced glide bands widely distributed over a large area (Supplementary Fig. 2c). However, the contrast of full dislocation is barely observed after deformation, indicating the dominant role of partial dislocations at larger size.

According to microstructural characterization, both full and partial dislocations mechanisms are concurrently triggered in submicron-scales by achieving sufficiently high stress. However, partial dislocation-mediated plasticity is rather limited due to limited mobility and extensive interactions. We observed evidence of partial dislocation activity on parallel, {111} glide planes in the form of twins and stacking faults which intersected with other partials and full dislocations from identical slip systems (Supplementary Fig. 2a and 2b). This kind of intersection forms locks which prevent further dislocation motion[1]. By contrast, a large number of full dislocations emitted from a single source can slip on the same {111} slip plane and accommodate larger plastic

strain. At larger sizes (2 $\leq D \leq$ 3.5 μm), the critical stress (~4 GPa) for full dislocation nucleation was not achieved in lithographic Si; favoring partial dislocation nucleation and emission. To accommodate plasticity in larger pillars, glide bands need to propagate to large areas, but then they are more likely to react with each other and form locks resulting in crack formation with further strain[1]. This limits the plasticity in large pillars. As larger sizes (5 μm $\leq D$), catastrophic fracture was observed in the pillars without measurable plasticity.

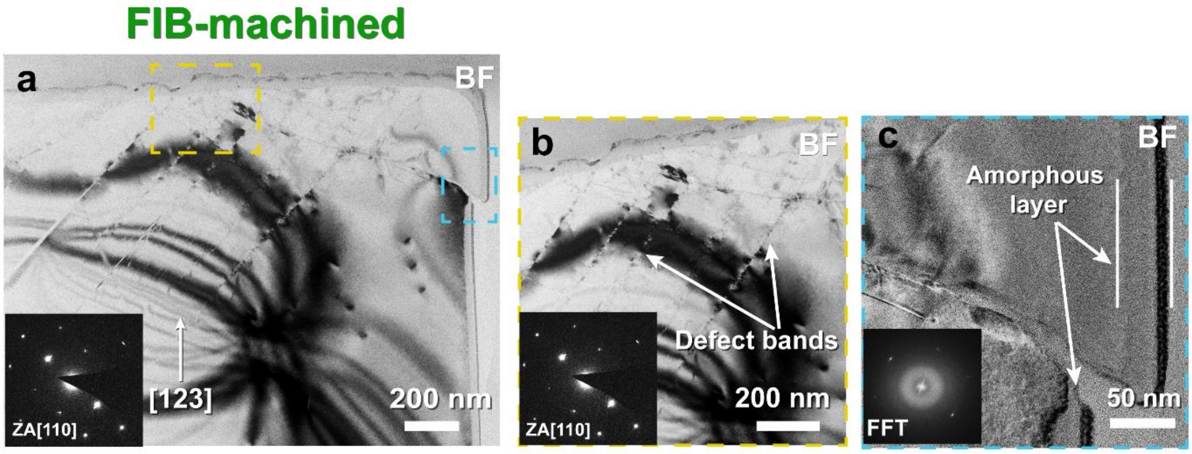

Figure 4. TEM bright-filed (BF) micrographs of ⟨123⟩-oriented Si pillar with a diameter of 1.5 μm from FIB milling deformed to 4% engineering strain at room temperature. **a** BF image of heavily deformed pillar top. **b** BF image of the local are at the pillar top with the glides band initiating from the interface between amorphous layer and top surface. **c** High resolution BF image of the crack at side surface with an amorphous layer with a thickness of ~40 nm, as indicated by the arrow between the solid lines.

The microstructure of the FIB-machined pillars was also inspected using the TEM after deformation (Supplementary Fig. 4). FIB-machined pillar attained an amorphous layer with a thickness of ~40 nm (Supplementary Fig. 4c) at the surface of pillar from Ga^+ irradiation. Glide bands initiated from the top interface below the amorphous layer (Supplementary Fig. 4b) and then propagated downwards. Since the top surface of Si pillar was irradiated by Ga^+ with 90° incident angle, extensive defects were implanted into the Si crystalline structure. Consequently, the top surface of pillar is irregular after FIB irradiation as shown in Supplementary Fig. 4a and 4b. These defects acted as the nucleation sites for dislocation during the subsequent plastic deformation.

Supplementary References

1. Howie PR, Korte S, Clegg WJ. Fracture modes in micropillar compression of brittle crystals. *Journal of Materials Research* **27**, 141-151 (2011).
